# Ramped position, an uncertain future

**DOI:** 10.1186/s13054-018-2045-6

**Published:** 2018-05-13

**Authors:** Luigi Vetrugno, Daniele Orso, Tiziana Bove

**Affiliations:** Anesthesiology and Intensive Care Clinic, Department of Medicine, University of Udine, 33100, P.le S. Maria della Misericordia 15, Udine, Italy

We have carried out a detailed reading of the article by Cabrini et al., “Tracheal intubation in critically ill patients: a comprehensive systematic review of randomized trials” [1], in which the authors conducted a comprehensive review of the effects of orotracheal intubation in critically ill patients. The article provides the most up-to-date, complete review on this topic. Evidence of the usefulness of pre-oxygenation is incontrovertible. However, we disagree with the authors’ conclusions regarding the ramped position. Specifically, we believe that the statement “the effect of the ramped position [results] in increasing the number of intubation attempts” is not sufficiently evidence based. Cabrini et al. refer to a single study—a randomized controlled trial by Semler et al. [2]—in which randomly selected ICU patients were intubated either in the sniffing position or in the ramped position. The primary outcome of this study was the improvement of the lowest saturation between the two groups. The authors did not highlight a statistically significant difference. Secondary outcomes were the grade of glottic view, the difficulty of intubation, and the number of intubation attempts. The authors observed worse results for these outcomes in the ramped position group. Xue et al. [3], however, have raised some questions about the reliability of this study. We would also like to point out that the conclusion reached by Cabrini et al. in their systematic review referred to a secondary outcome of the trial by Semler et al. The statistical power of the latter was calculated to respond to the primary end-point. However, the sample size was not adequate to respond incontrovertibly to the secondary outcomes. As secondary outcomes, the obtained results regarding the difficulty of intubation and the number of attempts can only be taken as hypothesis generators [4]. We believe, therefore, that the conclusion regarding the ramped position reported by Cabrini et al. is not sufficiently evidence based, and that questions remain as to its validity.
