# Supplementary material for: Ramped position, an uncertain future
Source: Crit Care. 2018 May 13;22:129. doi: 10.1186/s13054-018-2045-6 (PMC5949150; doi:10.1186/s13054-018-2045-6)
Supplement: Related texts

# Reply: Authors’ response

**Authors:** Luca Cabrini, Giovanni Landoni, Alberto Zangrillo, Massimo Antonelli, Rinaldo Bellomo, Paolo Pelosi

We are grateful to Vetrugno and co-workers for their careful reading of our article [1]. Their letter gives us the opportunity to further underline the two most relevant characteristics of our study.

First of all, we fully agree with the comments made by Vetrugno et al. on Semler’s randomized trial [2]: the evidence in favor of the sniffing position compared to the ramped position is weak, derived from secondary outcomes, and limited to the number of intubation attempts and quality of glottis view, while other outcomes showed no difference. On the other hand, the study was relatively large and multi-center, so increasing its reliability. We were very cautious in our conclusions, stating that “The sniffing position *might* be the position of choice for laryngoscopy.” Further research is clearly required before a strong recommendation can be made, and again (in line with Vetrugno’s et al. letter) we stated that “Our findings … will inform future research.” This was a main purpose of our study.

Second, we would underline that the results by Semler et al. are in contrast with previous studies performed in the operative theatre in elective patients [5–7]. This supports the assumption at the basis of our study that intubation of critically ill patients can be quite different from intubation of elective surgical patients in the operating theatre. Until new studies are published, the weak evidence from Semler et al.’s study is the best available evidence for critically ill patients.

In conclusion, in accordance with Vetrugno et al. we hope that in the near future more data will be made available to improve the effectiveness and safety of tracheal intubation in the critical care scenario.
